# Statistics-Based Outlier Detection and Correction Method for Amazon Customer Reviews

**DOI:** 10.3390/e23121645

**Published:** 2021-12-07

**Authors:** Ishani Chatterjee, Mengchu Zhou, Abdullah Abusorrah, Khaled Sedraoui, Ahmed Alabdulwahab

**Affiliations:** 1 Department of Electrical and Computer Engineering, New Jersey Institute of Technology, Newark, NJ 07102, USA; ic53@njit.edu; 2 Department of Electrical and Computer Engineering, Faculty of Engineering, and Center of Research Excellence in Renewable Energy and Power Systems, King Abdulaziz University, Jeddah 21481, Saudi Arabia; aabusorrah@kau.edu.sa (A.A.); sedraoui@yahoo.com (K.S.); aabdulwhab@kau.edu.sa (A.A.)

**Keywords:** sentiment analysis, interquartile range, TextBlob, natural language processing, outlier detection, data scrapping, J-shaped distribution, imbalance dataset, big data analytics

## Abstract

People nowadays use the internet to project their assessments, impressions, ideas, and observations about various subjects or products on numerous social networking sites. These sites serve as a great source to gather data for data analytics, sentiment analysis, natural language processing, etc. Conventionally, the true sentiment of a customer review matches its corresponding star rating. There are exceptions when the star rating of a review is opposite to its true nature. These are labeled as the outliers in a dataset in this work. The state-of-the-art methods for anomaly detection involve manual searching, predefined rules, or traditional machine learning techniques to detect such instances. This paper conducts a sentiment analysis and outlier detection case study for Amazon customer reviews, and it proposes a statistics-based outlier detection and correction method (SODCM), which helps identify such reviews and rectify their star ratings to enhance the performance of a sentiment analysis algorithm without any data loss. This paper focuses on performing SODCM in datasets containing customer reviews of various products, which are (a) scraped from Amazon.com and (b) publicly available. The paper also studies the dataset and concludes the effect of SODCM on the performance of a sentiment analysis algorithm. The results exhibit that SODCM achieves higher accuracy and recall percentage than other state-of-the-art anomaly detection algorithms.

## 1. Introduction

Sentiment analysis, emotion artificial intelligence, and intent analysis are often used to describe the same concept, i.e., opinion mining. Sentiment analysis uses a combination of natural language processing (NLP), computational linguistics, and text mining to analyze, derive, calibrate, and evaluate textual information in the form of sentences, phrases, documents, etc. [1]. NLP has earned a lot of attention recently.

People have started to rely on consumer reviews and sentiments shared over social media sites, blogs, and consumer feedback websites on the internet before purchasing or opting for a particular product or service. It has also become a vital tool for decision-makers who plan to improve, modify, or perform necessary actions based on public opinions.

Sentiment analysis is used extensively in various domains such as marketing, politics, sports, and stocks for information extraction, improvement of an automated chatbot response system, or product modification. Most companies use sentiment analysis to research consumer requirements and understand the market trends. Positive reviews of a product or service drive online marketing, while negative comments motivate companies to improve their products or services based on customer demands. Social media has become a robust platform that helps understand public opinions, acceptance, or issues regarding specific laws or lawmakers. Sentiment analysis helps one study the endorsement rate of these policies based on previous trends, which allows lawmakers to prepare and motivate the public accordingly. Similarly, this method aids in fan engagements and player/team reputation build-up in sports. It also helps one study a company’s prominence in the market, which impacts its stock valuation. These are some of the applications of sentiment analysis, to name a few.

With the expansion in data available through the internet, researchers have started focusing on both the academic and commercial applications of sentiment analysis. The boost in smartphone usage has increased the development of mobile games and apps. Oyebode et al. [2] used sentiment analysis to analyze the mental health apps in smartphones to classify their features as positive or negative. This analysis led to some design modifications based upon the negative factors of the app, which helped the app increase its potency. Afzaal et al. [3] used aspect-based sentiment analysis to implement a tourism app in smartphones to identify the most recommended restaurants and hotels in a city by extracting and classifying information from tourist reviews. In the fashion industry, online reviews play a vital role as it helps designers understand a shopper’s experience via the latter’s feedback. Li and Xu [4] proposed an aspect-based fashion recommendation model with an attention mechanism. They used convolutional neural networks, long short-term memory networks, and attention mechanisms to process customer and product reviews simultaneously. They then combined them to apprehend both local and global aspects of the reviews, which helps predict the customer rating.

Outlier detection is a salient data analysis concern that focuses on identifying oddities in datasets. Outlier (a.k.a. anomaly, noise, and exception) detection helps recognize an entity that prominently differs from most of the samples in a dataset [5]. Such entities may represent bank frauds, spam emails, structural defects, and errors in a dataset. Anomaly detection faces many challenges due to (a) the characteristic of input data or the nature of outliers, (b) noise in a dataset that might mimic an outlier, (c) inaccurate boundaries between standard data and outliers, and (d) computational complexity. In [6], Wang et al. explained the importance of designing an efficient and scalable outlier detection algorithm because the probability of the number of outliers is directly proportional to the volume of a dataset. It is also critical to promptly identify and rectify the outliers in a dataset such that we can have high-quality data.

The definition of an outlier may vary for various scenarios. For example, in this 5-star Amazon review for a hand sanitizer, “*Do not buy. Doesn’t sanitize for covid19. Does not contain alcohol. Fake description as sanitizer.*”, the nature of the review is positive as opposed to the sentiment of the review comment. Much like the example, this paper defines novelty as the reviews that have sentiment opposite to their corresponding star ratings. Anomaly detection is an eminently researched topic in various domains [7], but there is an inadequate study on outlier detection using sentiment analysis of a dataset. It is classified predominantly into supervised and unsupervised learning. The former is true when the dataset used is labeled, while the latter arises when the dataset is not labeled. The techniques used to identify anomalies are based broadly on classification, clustering, distance, machine learning, and statistical approaches. This paper proposes an outlier detection method using a combination of statistical and distance-based techniques. Our concerned dataset is scraped from the Amazon website, which consists of several Amazon products from various departments.

The rest of the paper structure is as follows: Section 2 reviews relevant sentiment analysis and outlier detection work. Section 3 discusses and analyzes the dataset used, and Section 4 presents the proposed statistics-based outlier detection and correction method (SODCM). Section 5 summarizes the experimental results. Section 6 showcases the conclusion and future work.

## 2. Related Work

Social media has become a powerful platform for people to share their opinions and concerns on topics ranging from socio-economic to political to technological advancements. Iglesias et al., in [8], discussed advancements in various approaches in the field of sentiment analysis, their contributions, and their applications in various domains. The work in [9] compiles all the studies related to various limitations of sentiment analysis on social media datasets. It discusses problems as trivial as spelling and grammatical mistakes to situations as critical as rumor-mongering, community shaming, riots, and protests arising from posts or comments on the internet. It also highlights the increasing impact of research conducted on sentiment analysis applied to social media datasets. The study in [10] analyzed previous literature based on modern social media applications. It also featured their impacts in healthcare, disaster management, and business.

In [11], Wang et al. explained that a sentence that holds an opinion consists of quintuple parameters (*e*, *a*, *s*, *h*, *t*), where *e* is the target or entity, *a* is the aspect or feature of *e*, *s* is the nature of the opinion on *e* or *a*, *h* is the opinion holder, and *t* is the time when *h* expresses the sentiment. For instance, in this 5-star Amazon review for a hand sanitizer, “*With having to use hand sanitizers so much due to the COVID situation, this is the best one I have found. Love the residual effects and the fact that is doesn’t dry out my skin. Would recommend over other brands.*”, *e* is the hand sanitizer, *a* is the residual effect, the nature of the opinion is positive, and the opinion holder is the Amazon reviewer while time is during COVID-19 pandemic. Sentiment analysis focuses explicitly on *s*, which is the nature of the opinion.

Sentiments or emotions tenaciously drive a consumer’s decisions and views regarding a product or service. The research in [12] focused on social media’s impact on people from a spatial and temporal vantage point. Using Alteryx, it filtered the tweets based on residential users from the 2016 United States Geo-tweets dataset. The results show a higher impact of tweets, especially those with positive sentiments, based on several features such as location, content, and time. Cosmetic brands apply sentiment analysis to obtain a clear and comprehensive insight into consumers’ thoughts on product quality and desires. In [13], Park implemented Term Frequency–Inverse Document Frequency to analyze the polarity of customer opinions and brand satisfaction for 26 different cosmetic companies. The research also focused on the factors affecting the nature of consumers’ views.

Understanding a consumer’s buying choices is a challenging assignment for a machine learning algorithm. Hu et al., in [14], introduced credibility, interest, and sentiment enhanced recommendation model, which consists of five segments, namely, feature extraction of the review, interest mining on the aesthetic of the comment, candidate feature sentiment assignment based on the nature of their fastText sentiment, and a recommendation module that utilizes credibility weighted sentiment score of the feature selected by the buyer and reviewer credibility evaluation that helps in weighing the credibility of the reviewer to avoid fake reviewers. The reviews also depend on a reviewer’s experience, which might differ from one customer to another. Li et al. focused on this problem in [15] by recommending an algorithm inspired by Dempster–Shafer’s evidence theory. They used hotel customer reviews of four different properties as a case study and extracted information from various travel websites to identify the practicability and capability of the algorithm. Their approach can help the managers develop strategies based on the customer reviews to outrun their competitors.

Aspect-based sentiment analysis (ABSA) identifies the feature/aspect of an entity/target in an opinion/review and then performs sentiment analysis on each element analyzed. In this 3-star Amazon review on gloves, “*Good value for the money, however, they do not hold up very well. They rip easily*”, the two aspects the consumer discusses are (a) affordability, whose sentiment is positive as they are cheap, and (b) durability, which carries a negative polarity. In [16], feature-focused sentiment analysis was applied to the customer comments, and the review votes of various mobile products were collected from Amazon. The result indicated that the method helps the manufacturers in product development and the buyers make a personalized decision based on multiple features of the product. Ali et al. [17] studied the customer reviews and feedbacks for ridesharing services to modify and uplift several organizations for Kansei engineering in India–Pakistan. Since the languages used commonly are Urdu/Hindi and English, the work converted all the reviews into English and performed ABSA. They also extracted the most frequently used aspect to improve further the services provided based on customer demands. ABSA also helps classify reviews or comments based on various product or service features related to the opinion. ABSA has several challenges, such as that the attention-based models may sometimes (a) lead to a given aspect incorrectly targeting grammatically irrelevant words, (b) fail to diagnose special sentence structures such as double negatives, and (c) weigh only one vector to depict context and target. In [18], Zhang et al. proposed a knowledge-guided capsule network to address the above limitations using Bi-LSTM and capsule attention network. The study in [19] summarizes the state-of-the-art ABSA methods by using lexicon-based, machine learning, and deep learning approaches.

In this digital age, since information is so readily available, before purchasing a product, buyers tend to read customer reviews and comments, which affect their purchasing decision. Researchers usually focus on the review body, but a review contains more information than that, which is generally not exploited, such as review time, number of helpful votes, review time, reviewer id, and review rating. In [20], Benlahbib and Nfaoui visualized the reputation of a product differently by considering all the parameters and projecting the reputation value, opinion category, top positive review, and top negative review. They implemented the time of review and the number of helpful votes for each review from the Transformers model to Bidirectional Encoder Representations. This helps to predict the probability of the nature of review sentiment. They also proposed equations that calculate the reputation value for a product. Extensive research is being conducted not only focusing on sentiment analysis in English but also several other languages such as Arabic [21], Persian [22], Urdu [23], Hindi [24], Russian [25], Chinese [26], and Indonesian [27].

Several studies were conducted on sentiment analysis [28] and its application on e-commerce. With the increase in online consumption, e-commerce enhancement has become a hot topic for research. Many scholars introduced methods focusing on deep neural networks [29], probabilistic classifiers [30], linear classifiers [31], lexicon-based approaches [32], or decision trees [33] to increase accuracy and efficiency. In [34], Wang et al. proposed an iterative sentiment analysis model called SentiDiff, which predicts polarities in Twitter messages by considering the interconnections between textual information of Twitter messages and sentiment diffusion patterns. Shofia and Abidi [35] used a support vector machine to identify the keywords and extricate the sentiment polarity of Twitter data specific to Canada on social distancing due to COVID-19. Zhang et al. [36] introduced a convolutional multi-head self-attention memory network to glean valuable and intricate semantic information from sequences and aspects of a sentence. This algorithm uses a convolutional network to capture n-gram grammatical knowledge and multi-head self-attention to acknowledge the linguistic information of the sequence by the memory network. Abdalgader et al. [37] applied a lexicon-based word polarity identification method by studying the semantic relatedness between the set of the target word and synonyms of words surrounding the target on several benchmark datasets. The result has outrun several existing methods that use pairwise relatedness between words at term-level around the target over a fixed size. The performance of various sentiment analysis methods differs due to such factors as datasets, feature representations, or classification processes. Liu et al. [19] conducted a detailed survey on several deep learning approaches for aspect-based sentiment analysis using benchmark datasets evaluation metrics and the performance of the existing deep learning methods.

Outliers are extreme values that diverge from the rest of the data samples [38,39]. It might occur due to an imbalanced dataset or experimental error, or novelty. The research [39] defines an outlier in its experiment as any tweet in a Twitter dataset that is not relevant to the topic in consideration. Once the outliers are detected and eliminated, it is noticed that the algorithm’s accuracy improves significantly. Similarly, in [40], it was observed that before implementing a convolutional neural network to the document to be classified, if outliers are identified and erased by using a density-based clustering algorithm, the efficiency increases, and the computational cost decreases. Kim et al. [41] applied a combination of four outlier detection methods, namely (a) Gaussian density estimation, (b) Parzen window density estimation, (c) Principal component analysis, and (d) K-means clustering to identify malicious activities in an institution using user log database. The outlier identification methods can be broadly categorized into statistical-based [42], distance-based [43], graph-based [44], clustering-based [45], density-based [46], and ensemble-based [47]. Once the outliers are detected, it is crucial to delete, consider, or modify the outlier. This usually depends on an outlier’s effect on the dataset if it is deleted or tampered with. The condition of an outlier can vary for different applications and datasets; for instance, if in a population estimation survey the number of people with height over 7 ft is very low, then these data can be verified and kept as they are natural outliers. In contrast, if in a dataset with various brands of shoes, the price of one or two are extraordinarily high, then those outliers can be deleted before calculating the average cost of a pair of shoes.

## 3. Datasets

With the advancement in the field of the internet and cloud computing [48], data collection has become more accessible. Public datasets are found in abundance for research purposes. Amazon is one of the many colossal data sources that encourage scholars to scrape publicly available data from their websites for research purposes. Based on a survey from Feedvisor, an article in Forbes concluded that 89% of the buyers choose Amazon instead of other e-commerce websites to make online purchases [49]. There are two types of datasets used in this paper, (a) collected datasets and (b) publicly available datasets. Collected datasets used in this paper [50] consists of product reviews we ourselves collected from Amazon.com, starting from the year 2008 to 2020, spanning across seven different domains, namely, book (Becoming by Michelle Obama), pharmaceutical (Turmeric Curcumin Supplement by Natures Nutrition), electronics (Echo Dot 3rd Gen by Amazon), grocery (Sparkling Ice Blue Variety Pack), healthcare (EnerPlex 3-Ply Reusable Face Mask), entertainment (Harry Potter: The Complete 8-Film Collection), and personal care (Nautica Voyage By Nautica).

**Figure 1:**
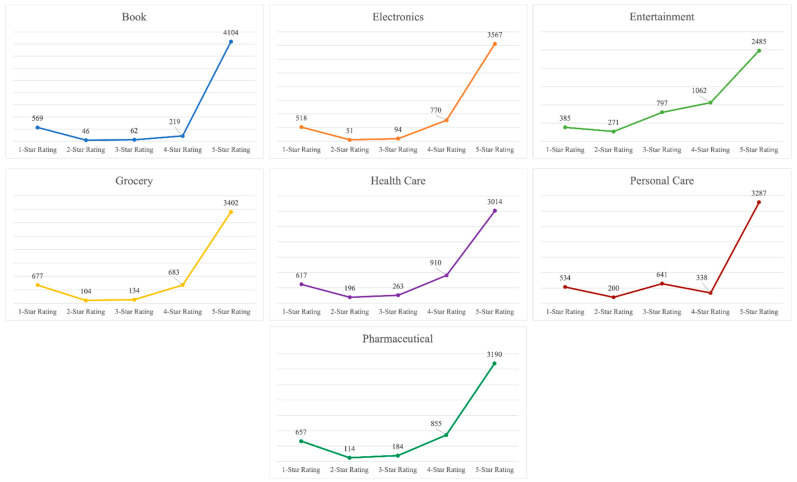
J-shaped distribution of the tallied reviews from all the accumulated datasets.

Each review carries multiple information such as reviewer name, date and place of comments, star rating, verified purchase, the number of buyers who find the review helpful, and the images added by the reviewer. This dataset scraped from Amazon consists of 35,000 Amazon customer reviews, including the product name, comment date, star rating, and the number of helpful votes. Figure 1 shows the number of reviews against each star rating accumulated for all seven collected datasets. It can be observed that the extremely positive star rating (5-star) dominates the dataset, and there are very few negative (1- and 2-star) and moderately positive (3- and 4-star) star ratings. The skewed nature of the dataset results in J-shaped distribution. Multiple reasons behind such bias towards extremely positive reviews exist. People usually agree with and write about the positive ratings and comments quickly but are generally skeptical about the negative ratings or comments. When a consumer notices an extremely positive review, it usually influences the consumer’s opinion resulting in the switching of star rating. A higher rating was also observed to easily influence a consumer to increase the valuation, while the reverse is not true [51]. Table 1 represents the consumer review distribution across the different star ratings in all the collected datasets individually. The results show the same biases of customer reviews towards a 5-star rating as compared to the rest.

**Figure 2:**
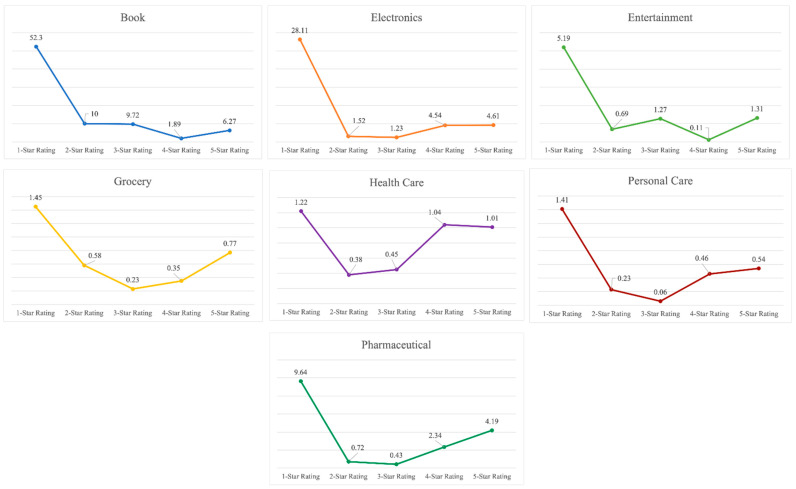
Average helpful votes per review across different star ratings.

Figure 2 represents a graphical distribution of the average number of helpful votes per review. It can be inferred that customers find the extremely negative reviews as the most helpful ones for making buying decisions or understanding a product. Extremely negative reviews are usually critical about the product, its features, packaging, delivery, usefulness, cost, and authenticity. It becomes easier for a consumer to decide about buying a product if they understand the various aspects of a product and the extremely negative experiences of former buyers. Table 2 compiles the average helpful vote per customer review in each dataset. It can be observed that most customers find extremely negative reviews most informative and beneficial.

## 4. Statistics-Based Outlier Detection and Correction Method (SODCM)

### 4.1. Interquartile Range

**Figure 3:**
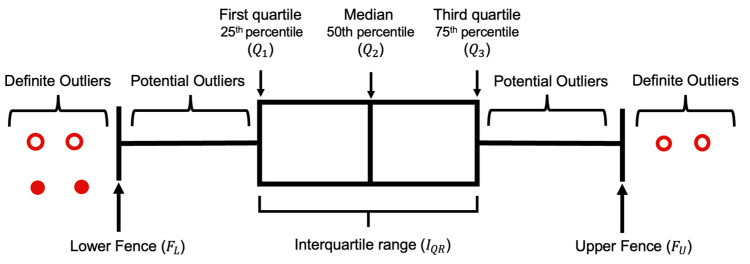
Box plot (with interquartile range) of a normal distribution for outliers’ detection.

Traditionally a dataset can be represented by using the five-number summary, which includes the lowest and highest value, median, and first and third quartile, the middle number between median and first and last number, respectively [52]. These values exhibit more information about a dataset as compared to just rows and columns. Figure 3 is an example of the box plot distribution of a dataset.

$Q_{1}$ and $Q_{3}$ are the intermediate points of the first and second half of an ordered dataset, respectively, and $Q_{2}$ is the median value of a dataset. For example, in an arranged dataset A={1,1,2,3,5,6,7}, $Q_{2}$ is 3, which is the median value or the fourth number of the dataset. $Q_{1}$ is 1 as it is the center value of the first half, 6 is $Q_{3}$ as it is the midpoint of the second half of the dataset.

The difference between $Q_{1}$ and $Q_{3}$ is the interquartile range ($I_{QR}$), which reflects the spread of the dataset about the median.

(1) $$I_{QR}=Q_{3}-Q_{1}$$

The lower and upper fences can be represented as:

(2) $$F_{L}=Q_{1}-1.5I_{QR}$$

(3) $$F_{U}=Q_{3}+1.5I_{QR}$$

Data in a dataset that exists beyond the bounds of $F_{L}$ and$\;F_{U}$ are outliers. Additionally, 1.5 preserves the sensitivity of the dataset. A larger scale than 1.5 would consider outliers as a datapoint, while the reverse would include data points in outliers.

In a dataset, there are two types of outliers, suspected or potential outliers and definite outliers. A potential outlier ($O_{P}$) is the data that are suspected as possible outliers if they satisfy:

(4) $$F_{L}<O_{P}<Q_{1}\;\;\mathrm{or}\;F_{U}<O_{P}<Q_{3}$$

A definite outlier ($O_{D}$) is the data that are absolute outliers if they comply with:

(5) $$O_{D}<F_{L}\;\mathrm{or}\;F_{U}<O_{D}$$

### 4.2. Definitions for SODCM

R consist of all the customer reviews in a dataset such that $R=\left\{r_{1},\;r_{2},\;r_{3},\ldots ,r_{N}\right\}$, where $r_{i}$ denotes $i^{th}$ review and $r_{i}^{*}$ is the star rating of $r_{i}$. In order to understand our proposed statistics-based outlier detection and correction method (SODCM), the following definitions are presented.

**Definition** **1.** $r_{i}$*is positive if*$r_{i}^{*}\;\geq 4$*, where*$r_{i}\in R$*. Any review with a star rating of four or more is considered a positive star rated review, denoted by*$S^{+}$.

**Definition** **2.** $\;r_{i}$*is negative if*$r_{i}^{*}<4$*, where*$r_{i}\in R$*. Any review with less than a four-star rating is considered a negative star rating review, denoted by*$S^{-}$.

**Definition** **3.** $\;T_{V}\left(r_{i}\right)=1\;if\;r_{i}\in S^{+}$*and*$T_{V}\left(r_{i}\right)=-1\;if\;r_{i}\in S^{-}$*. The target value of review*$r_{i}$*is 1 if it is a positive star rated review and −1 otherwise, denoted by*$T_{V}$.

**Definition** **4.**
$V_{D}\left(r_{i}\right)=d\left(T_{V}\left(r_{i}\right),\;C_{V}\left(r_{i}\right)\right)$ *, where* $C_{V}\left(r_{i}\right)$ *is the compound sentiment score of* $r_{i}$ *predicted by a sentiment analysis algorithm. The value difference of review* $r_{i}$ *is the Euclidean distance between* $T_{V}\left(r_{i}\right)$ *and* $C_{V}\left(r_{i}\right)$ *of the corresponding review, denoted by* $V_{D}\left(r_{i}\right)$ *. Since the range of both* $T_{V}$ *and* $C_{V}\;$ *is [−1, 1], the range of* $V_{D}$ *is [0, 2].*

### 4.3. Proposed Algorithm

The star rating assigned to a customer’s review is generally considered as the ideal sentiment of the comment. There are instances when a customer might have assigned a positive star review, but the nature of the feedback is negative. This 4-star Amazon customer review on a thermometer, “*Purchased the thermometer to have a method to check temperatures by non-contact. The thermometer’s box and content was not sealed which bothered me because of COVID*.”, carries a negative sentiment but has a positive rating which is contradictory. These ratings of reviews can be corrected to their correct star rating to improve the efficiency of a sentiment analysis algorithm.

**Figure 4:**
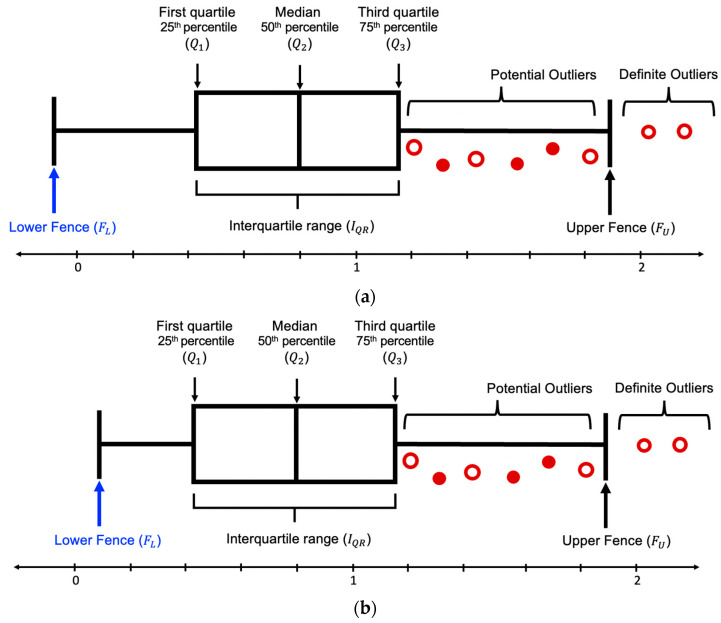
Box plot (with interquartile range) of *S*^+^ distribution for outliers’ detection. (**a**) depicts the box plot of *S*^+^ when *F*_*L*_ is negative and (**b**) depicts the box plot of *S*^+^ when *F*_*L*_ is positive.

**Figure 5:**
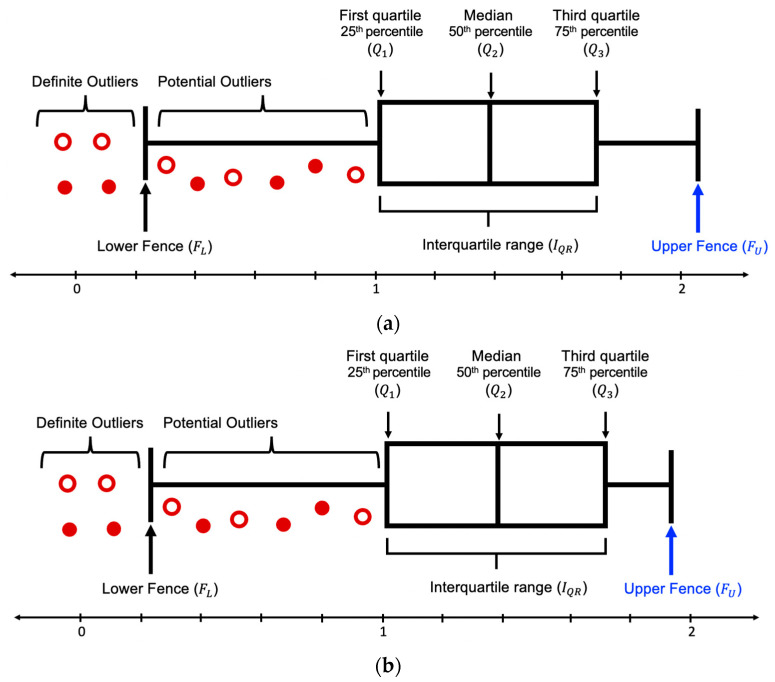
Box plot (with interquartile range) of *S*^−^ distribution for outliers’ detection. (**a**) depicts the box plot of *S*^−^ when *F*_*U*_ > 2 and (**b**) depicts the box plot of *S*^−^ when *F*_*U*_ ≤ 2.

SODCM consists of two major parts, namely the (a) detection of these outliers and (b) correction of these identified anomalies. It has the following six steps:

*Input:*  The input for SODCM is any dataset containing customer reviews ($r_{i}$) and their corresponding star ratings ($r_{i}^{*}$);
*Step 1:*  $T_{V}$ is calculated using $r_{i}^{*}$. If $r_{i}$ belongs to $S^{+}$ then $T_{V}=1$ and if $r_{i}$ belongs to $S^{-}$ then $T_{V}=-1$. Since this work focuses on the binary classification of the sentiments of customer reviews, the values assigned to $T_{V}$ are −1 or 1;
*Step 2:*  $V_{D}$ is calculated between $T_{V}$ and $C_{V}$. The value of $V_{D}$ is always positive. Since the minimum and maximum value $T_{V}$ and $C_{V}$ is 0 and 1, the range of $V_{D}$ is between 0 and 2. Figure 4 is an example of the box plot distribution of $S^{+}$. Since the minimum value $V_{D}$ can hold is 0, Figure 4a depicts the box plot of $S^{+}$ when $F_{L}$ is negative and Figure 4b depicts the box plot of $S^{+}$ when $F_{L}$ is positive. Figure 5 is an example of the box plot distribution of $S^{-}$. Since the maximum value $V_{D}$ can hold is 2, Figure 5a depicts the box plot of $S^{-}$ when $F_{U}$ > 2 and Figure 5b depicts the box plot of $S^{-}$ when $F_{U}$ ≤ 2;
*Step 3:*  After analyzing the dataset, it can be construed that $S^{+}$ has some reviews whose sentiment does not match the nature of star rating; hence, they are considered outliers. On the other hand, $S^{-}$ has very few reviews whose opinions match the essence of their respective star rating; hence, the reviews which are correctly assigned to their corresponding star ratings are considered outliers. This implies that most negative comments are incorrectly rated; therefore, the outliers, in this case, would be the correctly rated comments. In other words, the incorrectly labeled reviews are all the reviews in $S^{-}$, excluding the ones which are outliers. Hence, the dataset is split into $S^{+}$ and $S^{-}$;
*Step 4:*  In $S^{+}$, if $F_{L}$ is negative, then $O_{s}$ can be calculated as $Q_{3}+I_{QR}$ else, $F_{U}-I_{QR}F_{L}$. Since the range of $V_{D}$ is [0, 2], the least value it can hold is 0. In $S^{-}$ if $F_{U}>2$, then $O_{s}$ can be calculated as $Q_{1}-I_{QR},\;$else, $Q_{3}-I_{QR}F_{U}$. We compute $O_{s}$ as follows:
  (6) $$\mathrm{For}\;S^{+}\;\;\;\;\;O_{s}=\{\begin{array}{c} Q_{3}+I_{QR},\;\;F_{L}<0\\ \;\;F_{U}-I_{QR}F_{L},\;\;F_{L}\geq 0 \end{array}$$
  (7) $$\mathrm{For}\;S^{-}\;\;\;\;\;O_{s}=\{\begin{array}{c} Q_{1}-I_{QR},\;\;F_{U}>2\\ \;\;Q_{3}-I_{QR}F_{U},\;\;F_{U}\leq 2 \end{array}$$
*Step 5:*  In $S^{+}$, $V_{D}\left(r_{i}\right)\geq O_{s}$, if $r_{i}$ is outlier. For $S^{+}$, customer comments, whose $V_{D}\left(r_{i}\right)\geq O_{s}$, are outliers. In $S^{-}$, if $V_{D}\left(r_{i}\right)\leq O_{s}$, if $r_{i}$ is outlier. For $S^{-}$, customer comments whose $V_{D}\left(r_{i}\right)\leq O_{s}$, are outliers. These five steps complete the outlier detection process;
*Step 6:*  $T_{V}$ of reviews labeled as outliers in $S^{+}$ is reversed, meaning a comment with $T_{V}=1$ now becomes re-labeled as −1 and vice versa. On the contrary, for $S^{-}$, $T_{V}$ of reviews that are not labeled as outliers is reversed. This step is vital as it performs outlier correction by changing the nature of $r_{i}^{*}$;
*Output:*  The output of the proposed algorithm is the dataset consisting of reviews with their corrected nature of star ratings which means a positive natured review is labeled as 1 and the negative natured review as −1. SODCM helps in detecting the outliers and correcting them without eliminating or modifying any review.

The above steps are realized in SODCM. After its execution, we can perform a more accurate sentiment analysis of the revised dataset, and the obtain performance matrix of SODCM is obtained.

**Theorem** **1.** *The time complexity of SODCM is*O(n).

**Proof.**  Each of Steps 1 to 6 requires time complexity O(n)  while Step 4 needs O(1). Hence, the entire algorithm (Algorithm 1) has the complexity O(n). □

**Algorithm 1** Statistics-based outlier detection and correction method (SODCM)**Input:** D // dataset containing $r_{i}$ and $r_{i}^{*}$ Output: $D^{*}$// modified dataset post outlier detection and correction**Step 1:** 1 **if** $r_{i}^{*}\;\geq 4$ **then** 2** **$T_{V}=1$; 3 **else** 4 $T_{V}=-1$; 5 **end if** **Step 2:** 6 INITIALIZE $V_{D}$ to array [0]; 7 **for** each $r_{i}$ **do** 8 $V_{D}\left[i\right]=d^{E}\left(T_{V},\;C_{V}\right)$; 9 **end for** **Step 3:** 10 INITIALIZE $S^{+}$ to array [0]; 11 INITIALIZE $S^{-}$ to array [0]; 12 **for** each $r_{i}^{*}$ **do** 13 **if** $r_{i}^{*}\;\geq 4$ **then** 14 $S^{+}\left[i\right]=\left[r_{i},\;r_{i}^{*},V_{D}\left[i\right]\right]$; 15 **else** 16 $S^{-}\left[i\right]=\left[r_{i},\;r_{i}^{*},V_{D}\left[i\right]\right]$; 17 **end if** 18 **end for** **Step 4:** 19 **Function** $I_{QR}$ calculation $\left(S,V_{D}\right)$ 20 Sort $\left(\;V_{D}\right)$; 21 Let $\;Q_{1}=$ first quartile ($\;V_{D}$); 22 Let $\;Q_{3}=$ third quartile ($\;V_{D}$); $I_{QR}=Q_{3}-Q_{1}$; 23 $F_{L}=Q_{1}-1.5I_{QR}$; 24 $F_{U}=Q_{3}+1.5I_{QR}$; 25 **if** $\;S\;\geq \;S^{+}$ **then** 26 **if** $F_{L}<0$ **then** 27 $O_{s}=Q_{3}+I_{QR}$; 28 **else** 29 $O_{s}=F_{U}-I_{QR}F_{L}{;}_{}$ 30 **end if** 31 **Else** 32 **if** $F_{U}>2$ **then** 33 $O_{s}=Q_{1}-I_{QR}$; 34 **else** 35 $O_{s}=Q_{3}-I_{QR}F_{U}{;}_{}$ 36 **end if**37     **end if** 38 **return** $O_{s}$; 39 **end Function** 40 $O_{S+}=$ calculation $\left(\;S^{+},V_{D}\right)$; 41 $O_{S-}=$ calculation $\left(\;S^{-},V_{D}\right)$; **Step 5:** 42 INITIALIZE $O^{+}$ to array [0]; 43 INITIALIZE $O^{-}$ to array [0]; 44 **for** each $r_{i}$ in $\;S^{+}$ **do** 45 **if** $\;V_{D}\left(r_{i}\right)$ ≥ $O_{S+}$ **then** 46 $O^{+}\left[i\right]=$ ‘yes’; 47 **else** 48 $O^{+}\left[i\right]=$ ‘no’; 49 **end if** 50 **end for** 51 **for** each $r_{i}$ in $\;S^{-}$ **do** 52 **if** $\;V_{D}\left(r_{i}\right)$ ≤ $O_{S-}$ **then** 53 $O^{-}\left[i\right]=$ ‘yes’; 54 **else** 55 $O^{-}\left[i\right]=$ ‘no’; 56 **end if** 57 **end for** **Step 6:** 58 **for** each $r_{i}$ in $\;S^{+}$ **do** 59 **if** $O^{+}\left[i\right]=$‘yes’ **then** 60 $T_{V}\left[i\right]=$ toggle$\left(T_{V}\left[i\right]\right)$; 61 **end if** 62 **end for** 63 **for** each $r_{i}$ in $\;S^{-}$ **do** 64 **if** $O^{-}\left[i\right]=$‘no’ **then** 65 $T_{V}\left[i\right]=$ toggle$\left(T_{V}\left[i\right]\right)$; 66 **end if** 67 **end for** 68 $D^{*}=$ concat $\left(\;S^{+},S^{-}\right)$;

## 5. Experimental Results

The proposed SODCM identifies and rectifies outliers for all the datasets consisting of Amazon customer reviews of products from various domains. All the three algorithms are executed on both (a) collected Amazon review datasets and (b) an Amazon review dataset publicly available in the amazon-reviews-pds S3 bucket in AWS US East Region [53]. There are several datasets consisting of product reviews from various domains, and we chose Amazon product review datasets for seven domains, namely apparel, beauty, fashion, furniture, jewelry, luggage, and toys. Each of these datasets consists of 100,000 customer reviews. The algorithm used for sentiment analysis is TextBlob [54], which is a Python library for NLP. The experiment is performed in two stages. Initially, the algorithm is implemented to each star rating of a dataset separately to study the results. SODCM then evaluates the complete dataset at a later stage of the research.

Table A1, Table A2, Table A3, Table A4 and Table A5 in Appendix A represent the results from reviews evaluated based on the star ratings individually. For Table A1 and Table A2, the least value for $O_{s}$ is considered as $F_{U}$, and $O_{s}$ is then decremented by 0.1 until it reaches 0.8. For Table A3, Table A4 and Table A5, the least value for $O_{s}$ is considered as $F_{L}$, and $O_{s}$ is then incremented by 0.1 until it reaches 1.2. The results are then saved in a csv file, evaluated manually to check the number of outliers detected correctly and incorrectly. In all the Tables, $O_{D}$ represents the total number of outliers detected, $O_{I}$ is the number of reviews incorrectly labeled as outliers, and $O_{C}$ equals the number of reviews correctly labeled as outliers. $O_{I}$ and $O_{C}$ are validated manually for cross-verification. SODCM is implemented for all the datasets and ratings separately.

The performance of SODCM is compared with two state-of-the-art outlier detection methods published this year: (a) a class-based approach [55] and (b) a deep-learning-based approach [56]. Table 3 and Table 4 represent the performance comparison of SODCM with those in [55,56] on the collected datasets and on the publicly available datasets, respectively. The bold numbers in all tables mean the best results among three methods. Table 5 compiles the metrics comparison for SODCM using *p*-value, T-score, and CI, where CI represents the 95% confidence interval in the form of [*x*, *y*].

From Table A1, Table A2, Table A3, Table A4 and Table A5, it can be concluded that SODCM detects an optimal number of outliers in all the datasets and shows a perfect ratio between the correctly and incorrectly detected outliers, thus resulting in a high degree of accuracy. The accuracy decreases considerably once the value of $O_{s}$ reaches one. Moreover, the increase or decrease in $O_{s}$ for positive or negative star-rated reviews, respectively, results in a rise in incorrectly labeled outliers. It can also be concluded from Table 3 and Table 4 that the accuracy and recall percentage of SODCM for all the datasets outperform those of [55,56]. Hence, it is inferred that SODCM outperforms the other methods in the outlier detection and correction approach, which are outperformed by those in [55,56].

Table 5 reflects that the *p*-value is less than 0.001, which is robust evidence against the null hypotheses. An extremely low *p*-value signifies that the results are not accidental, and the improvement is due to SODCM. The T-score for all the datasets is high, indicating more significant evidence against the null hypothesis. This means that there is a considerable difference between the collected star ratings from the website and the improved star ratings based on the nature of the reviews by SODCM. CI in Table 4 represents a 95% chance that the actual error of the model is between *x* ± *y*. Hence, the smaller CI, the more precise is the estimate of the model.

## 6. Conclusions and Future Work

SODCM is a novel approach tor identifying anomaly in a customer review dataset and rectifying it by improving their corresponding star rating. The results exhibit that the performance of the proposed algorithm surpasses other state-of-the-art approaches, and it also gives evidence for SODCM’s rejection of the null hypothesis. The advantage of SODCM against most of the methods is that this data analysis pipeline preserves the outliers to correct them and prevents any information loss. From this dataset study, it can also be inferred that the outlier definition is different for positive and negative reviews as the minority in a dataset with positive star rated reviews is when the nature of both reviews and star ratings contradicts. At the same time, the reverse is true for negative star-rated reviews. Moreover, Amazon customer review datasets are generally highly imbalanced irrespective of the product or its department, and they follow J-shaped distribution. By studying the count of helpful votes in the datasets, it is noticed that extremely negative reviews are the most critical ones, which help in the decision-making for the majority of the customers.

Since it can be concluded that SODCM performs well on datasets consisting of Amazon customer reviews, the future work should focus on applying the proposed method to product reviews from other marketplace datasets such as eBay, Etsy, Best Buy, Target, Walmart, etc., to obtain a better insight into the discrepancies between star ratings and the related reviews. This will help conclude that SODCM can detect and rectify anomalies without deleting any data to preserve the overall dataset knowledge. This algorithm can be implemented in several real-life scenarios such as accessing product performance [57,58,59,60,61,62], conducting market research along with flagging of reviews through rating and review irregularity detection, and thus rectifying them without any data loss [63,64]. In this paper, the sentiment analysis algorithm used is TextBlob, a Python-based NLP package. It should be interesting to study the behavior and impact of SODCM when combined with other state-of-the-art sentiment analysis algorithms such as BERT, XLNet, ELECTRA, OpenAI’s GPT-3, RoBERTa, or StructBERT.

## Figures and Tables

**Table 1:** Review distribution across different star ratings.

Dataset	5-Star Rating	4-Star Rating	3-Star Rating	2-Star Rating	1-Star Rating
Book	4104	219	62	46	569
Electronics	3567	770	94	51	518
Entertainment	2485	1062	797	271	385
Grocery	3402	683	134	104	677
Health Care	3014	910	263	196	617
Personal Care	3287	338	641	200	534
Pharmaceutical	3190	855	184	114	657

**Table 2:** Average helpful votes per review across different star ratings.

Dataset	5-Star Rating	4-Star Rating	3-Star Rating	2-Star Rating	1-Star Rating
Book	6.27	1.89	9.72	10	52.3
Electronics	4.61	4.54	1.23	1.52	28.11
Entertainment	1.31	0.11	1.27	0.69	5.19
Grocery	0.77	0.35	0.23	0.58	1.45
Health Care	1.01	1.04	0.45	0.38	1.22
Personal Care	0.54	0.46	0.06	0.23	1.41
Pharmaceutical	4.19	2.34	0.43	0.72	9.64

**Table 3:** Performance comparison of SODCM with state-of-the-art approaches.

Dataset	Methods	Accuracy%	Recall%	$O_{D}$
Book	**SODCM**	**96.9**	**98.4**	**75**
	[55]	84.1	52.2	410
	[56]	86.1	50.2	955
Electronics	**SODCM**	**93.1**	**96.5**	**60**
	[55]	67.3	49.8	193
	[56]	71.3	48.5	638
Entertainment	**SODCM**	**87.6**	**93.8**	**23**
	[55]	67.7	51.8	158
	[56]	79.1	48.9	1434
Grocery	**SODCM**	**92.3**	**96.1**	**31**
	[55]	75.7	49.7	406
	[56]	85.8	48.1	1194
Health Care	**SODCM**	**93.1**	**96.5**	**43**
	[55]	74.8	51.1	99
	[56]	86.2	49.1	1025
Personal Care	**SODCM**	**93.3**	**96.6**	**31**
	[55]	76.3	50.9	717
	[56]	86.2	48.9	1177
Pharmaceutical	**SODCM**	**89.4**	**94.7**	**17**
	[55]	78.7	51.0	239
	[56]	77.3	47.2	971

**Table 4:** Performance comparison of SODCM with state-of-the-art methods on public datasets.

Dataset	Methods	Accuracy%	Recall%	$O_{D}$
Apparel	**SODCM**	**89.1**	**94.5**	**809**
	[55]	78.8	65.3	6404
	[56]	80.1	65.3	585
Beauty	**SODCM**	**90.4**	**95.1**	**936**
	[55]	81.2	65.4	9501
	[56]	83.1	65.5	643
Fashion	**SODCM**	**92.3**	**96.1**	**1061**
	[55]	81.6	62.2	3257
	[56]	81.4	62.1	604
Furniture	**SODCM**	**90.8**	**95.3**	**922**
	[55]	80.4	64.8	3743
	[56]	81.2	64.1	675
Jewelry	**SODCM**	**91.3**	**95.6**	**700**
	[55]	81.2	64.4	6345
	[56]	82.4	64.4	562
Luggage	**SODCM**	**92.1**	**96.2**	**831**
	[55]	82.1	63.6	4000
	[56]	83.3	63.8	599
Toy	**SODCM**	**90.2**	**95.1**	**662**
	[55]	83.2	65.7	9444
	[56]	84.1	65.2	634

**Table 5:** Metrics comparison for SODCM.

Dataset	*p*-Value	T-Score	CI
Book	1.77 × 10^−9^	9.05	[0.02, 0.04]
Electronics	1.43 × 10^−6^	16.67	[0.06, 0.08]
Entertainment	8.46 × 10^−8^	25.67	[0.11, 0.13]
Grocery	1.48 × 10^−7^	18.93	[0.07, 0.08]
Health Care	7.26 × 10^−6^	17.27	[0.06, 0.08]
Personal Care	1.08 × 10^−6^	17.38	[0.06, 0.07]
Pharmaceutical	3.62 × 10^−9^	23.63	[0.10, 0.12]

## Data Availability

This research uses two types of dataset a) collected dataset which we scraped from Amazon website and b) publicly available dataset. The collected data is available in a publicly accessible repository. The collected data presented in the study are openly available in Harvard Dataverse at doi/10.7910/DVN/W96OFO. The publicly available Amazon customer review data is available in TSV files in the amazon-reviews-pds S3 bucket in AWS US East Region.

## Appendix A

This Appendix projects all the experimental tables to support the results of this work. All the experiments were conducted in Python 3.7 on a Jupyter notebook. The models were tested locally on an Apple M1 chip, 8 GB of RAM, and 512 GB SSD. Several Python libraries were used including NLTK 3.5, pandas 1.2.0, matplotlib 3.3.3, TextBlob 0.15.3, scikit-learn 0.19.0, NumPy 1.19.5, scipy 1.7.1, and pingouin 0.4.0. For Table A1 and Table A2, the value of $O_{s}$ ranges between $F_{U}$ and 0.8 with a gradual decrement in steps of 0.1. For Table A3, Table A4 and Table A5, the value of $O_{s}$ ranges between $F_{L}$ and 1.2 with a gradual increment in steps of 0.1. The results, saved in a csv file, are manually evaluated twice by two different analysts to determine the number of outliers detected correctly and incorrectly. In all the Tables, $O_{D}$ represents the total number of outliers detected, $O_{I}$ is the number of reviews incorrectly labeled as outliers, and $O_{C}$ equals the number of reviews correctly labeled as outliers. $O_{I}$ and $O_{C}$ are validated manually for cross-verification.

From Table A1, Table A2, Table A3, Table A4 and Table A5, it can be observed an optimal number of outliers is successfully detected in all the datasets by the proposed SODCM. This leads to a high degree of accuracy since the number of correctly and incorrectly detected outliers reach a perfect balance. As the value of $O_{s}$ reaches 1, the sentiment analysis accuracy of the modified dataset decreases considerably, and the increase and decrease in $O_{s}$ for positive and negative star-rated reviews, respectively, results in a rise in incorrectly labeled outliers.

**Table A1:** SODCM applied to 5-star review comments.

Dataset	$O_{s}$	$O_{D}$	$O_{I}$	$O_{C}$	Accuracy
Book	1.181	55	9	46	0.973
	**1.141**	**74**	**17**	**57**	**0.978**
	1.1	92	26	66	0.982
	1.0	297	87	210	0.967
	0.9	485	109	376	0.922
	0.8	1005	129	826	0.795
Electronics	1.184	35	1	34	0.929
	**1.105**	**75**	**5**	**70**	**0.946**
	1.1	86	8	78	0.951
	1.0	231	154	231	0.991
	0.9	573	298	275	0.853
	0.8	1178	435	743	0.61
Entertainment	1.747	15	1	14	0.886
	1.7	17	2	15	0.887
	1.6	26	4	22	0.89
	1.5	40	9	31	0.894
	**1.478**	**43**	**9**	**34**	0.895
	1.4	65	13	52	0.902
	1.3	94	15	79	0.911
	1.2	147	21	126	0.926
	1.1	257	46	211	0.96
	1.0	705	351	354	0.903
	0.9	937	454	483	0.832
	0.8	1224	590	634	0.745
Grocery	1.6	25	1	24	0.924
	1.5	32	2	30	0.926
	1.4	45	4	41	0.93
	**1.355**	**54**	**4**	**50**	**0.933**
	1.3	71	10	61	0.937
	1.2	102	17	85	0.947
	1.1	162	35	127	0.964
	1.0	604	245	359	0.905
	0.9	774	301	473	0.855
	0.8	1061	354	707	0.771
Health Care	1.365	26	3	23	0.938
	**1.345**	**28**	**3**	**25**	**0.939**
	1.3	33	7	26	0.941
	1.2	76	12	64	0.954
	1.1	119	17	102	0.969
	1.0	400	183	217	0.937
	0.9	672	291	381	0.847
	0.8	1075	408	667	0.713
Personal Care	1.687	17	1	16	0.934
	1.6	21	1	20	0.935
	1.5	45	3	42	0.942
	**1.425**	**50**	**3**	**47**	**0.945**
	1.4	60	9	51	0.947
	1.3	78	9	69	0.953
	1.2	100	16	84	0.96
	1.1	161	43	118	0.979
	1.0	671	233	438	0.861
	0.9	801	295	506	0.82
	0.8	1039	375	664	0.745
Pharmaceutical	1.75	13	1	12	0.896
	1.7	14	1	13	0.897
	1.6	28	4	14	0.901
	**1.5**	**38**	**7**	**31**	**0.903**
	1.4	48	15	33	0.906
	1.3	77	28	49	0.914
	1.2	130	57	73	0.929
	1.1	242	114	128	0.961
	1.0	1203	146	1057	0.769
	0.9	1459	177	1282	0.697
	0.8	1744	207	1616	0.595

**Table A2:** SODCM applied to 4-star review comments.

Dataset	$O_{s}$	$O_{D}$	$O_{I}$	$O_{C}$	Accuracy
Book	1.174	5	1	4	0.981
	**1.138**	**6**	**1**	**5**	**0.986**
	1.1	6	1	5	0.986
	1.0	14	6	8	0.977
	0.9	26	8	18	0.922
	0.8	49	12	37	0.817
Electronics	1.194	9	2	7	0.928
	**1.114**	**21**	**2**	**19**	**0.939**
	1.1	23	4	19	0.941
	1.0	94	27	67	0.991
	0.9	260	122	138	0.835
	0.8	522	205	317	0.588
Entertainment	1.587	1	0	1	0.872
	1.5	4	0	5	0.881
	1.4	5	0	5	0.884
	**1.365**	**5**	**0**	**5**	**0.884**
	1.3	13	0	13	0.908
	1.2	19	0	19	0.926
	1.1	30	1	29	0.958
	1.0	73	18	55	0.914
	0.9	95	33	62	0.849
	0.8	127	52	75	0.754
Grocery	1.568	4	0	4	0.918
	1.5	7	0	7	0.922
	1.4	12	1	11	0.929
	**1.326**	**14**	**1**	**13**	**0.932**
	1.3	15	1	14	0.934
	1.2	22	6	16	0.944
	1.1	39	10	29	0.969
	1.0	115	62	53	0.919
	0.9	148	69	79	0.871
	0.8	198	82	116	0.797
Health Care	1.312	9	1	8	0.927
	**1.269**	**13**	**1**	**12**	**0.931**
	1.2	21	2	19	0.94
	1.1	44	9	35	0.965
	1.0	133	54	79	0.936
	0.9	214	93	121	0.847
	0.8	361	125	236	0.685
Personal Care	1.69	7	0	7	0.943
	1.6	7	0	7	0.943
	1.5	13	2	11	0.95
	**1.429**	**14**	**2**	**12**	**0.952**
	1.4	15	2	13	0.953
	1.3	18	3	15	0.956
	1.2	25	4	21	0.964
	1.1	41	6	35	0.983
	1.0	184	123	61	0.849
	0.9	213	147	66	0.815
	0.8	264	187	77	0.755
Pharmaceutical	1.75	1	0	1	0.894
	1.7	2	0	2	0.896
	1.6	4	1	3	0.898
	**1.5**	**5**	**1**	**4**	**0.9**
	1.4	8	3	5	0.903
	1.3	13	4	9	0.91
	1.2	26	9	17	0.927
	1.1	44	17	27	0.951
	1.0	243	139	104	0.79
	0.9	290	153	137	0.729
	0.8	383	161	222	0.609

**Table A3:** SODCM applied to 3-star review comments.

Dataset	$O_{s}$	$O_{D}$	$O_{I}$	$O_{C}$	Accuracy
Book	0.834	2	1	1	0.951
	0.9	3	1	2	0.967
	1.0	6	1	5	0.983
	**1.006**	**6**	**1**	**5**	**0.983**
	1.1	9	4	5	0.935
	1.2	16	10	6	0.822
Electronics	0.817	8	1	7	0.933
	0.9	24	6	18	0.953
	**0.993**	**59**	**8**	**51**	**0.997**
	1.0	65	11	54	0.994
	1.1	179	79	100	0.851
	1.2	376	210	166	0.604
Entertainment	0.279	4	0	4	0.87
	0.3	4	0	4	0.87
	0.4	5	0	5	0.872
	0.5	8	0	8	0.876
	**0.530**	**9**	**0**	**9**	**0.878**
	0.6	14	1	13	0.886
	0.7	16	1	15	0.889
	0.8	29	3	26	0.909
	0.9	46	7	39	0.936
	1.0	146	65	81	0.907
	1.1	210	90	120	0.808
	1.2	279	143	136	0.7
Grocery	0.572	1	1	0	0.902
	0.6	1	1	0	0.902
	0.7	2	1	1	0.91
	**0.771**	**4**	**1**	**3**	**0.925**
	0.8	5	2	3	0.932
	0.9	10	4	6	0.97
	1.0	21	12	9	0.947
	1.1	29	18	11	0.888
	1.2	40	26	14	0.805
Health Care	0.615	4	1	3	0.889
	0.7	5	1	4	0.893
	0.8	9	**1**	**8**	0.908
	**0.812**	**9**	**1**	**8**	**0.908**
	0.9	18	7	11	0.942
	1.0	51	24	27	0.931
	1.1	68	32	36	0.866
	1.2	113	66	47	0.695
Personal Care	0.241	2	0	2	0.902
	0.3	2	0	2	0.902
	0.4	2	0	2	0.902
	0.5	3	0	3	0.907
	**0.53**	**3**	**0**	**3**	**0.907**
	0.6	7	1	6	0.929
	0.7	9	2	7	0.94
	0.8	12	2	10	0.956
	0.9	16	5	11	0.978
	1.0	40	18	22	0.891
	1.1	46	22	24	0.858
	1.2	59	33	26	0.788
Pharmaceutical	0.482	1	0	1	0.893
	0.5	1	0	1	0.893
	0.6	1	0	1	0.893
	0.7	1	0	1	0.893
	**0.701**	**1**	**0**	**1**	**0.893**
	0.8	2	1	1	0.904
	0.9	8	3	5	0.989
	1.0	37	25	12	0.723
	1.1	44	31	13	0.648
	1.2	62	46	16	0.457

**Table A4:** SODCM applied to 2-star review comments.

Dataset	$O_{s}$	$O_{D}$	$O_{I}$	$O_{C}$	Accuracy
Book	0.793	1	0	1	0.978
	0.9	2	0	2	1
	**0.9706**	**2**	**0**	**2**	**1**
	1.0	4	1	3	0.956
	1.1	7	3	4	0.891
	1.2	17	10	7	0.673
Electronics	0.827	5	1	4	0.933
	0.9	11	3	8	0.955
	1.0	21	5	16	0.992
	**1.001**	**25**	**6**	**19**	**0.993**
	1.1	61	22	39	0.859
	1.2	124	65	59	0.627
Entertainment	0.346	1	0	1	0.82
	0.4	1	0	1	0.82
	0.5	1	0	1	0.82
	0.566	2	0	2	0.825
	0.6	2	0	2	0.825
	0.7	2	0	2	0.825
	0.8	8	1	7	0.855
	0.9	23	3	20	0.93
	1.0	50	13	37	0.935
	1.1	68	18	50	0.845
	1.2	95	31	64	0.71
Grocery	0.342	1	0	1	0.894
	0.4	2	1	1	0.903
	0.5	2	1	1	0.903
	**0.585**	**2**	**1**	**1**	**0.903**
	0.6	3	2	1	0.913
	0.7	4	2	2	0.923
	0.8	7	2	5	0.951
	0.9	8	2	6	0.961
	1.0	22	7	15	0.903
	1.1	29	12	17	0.836
	1.2	38	17	21	0.75
Health Care	0.599	1	0	1	0.887
	0.7	2	0	2	0.892
	**0.798**	**6**	**0**	**6**	**0.913**
	0.8	7	1	6	0.918
	0.9	13	4	9	0.948
	1.0	41	20	21	0.908
	1.1	58	29	29	0.821
	1.2	91	55	36	0.653
Personal Care	0.512	1	0	1	0.947
	0.6	1	0	1	0.947
	0.7	1	0	1	0.947
	**0.734**	**1**	**0**	**1**	**0.947**
	0.8	1	0	1	0.947
	0.9	4	0	4	0.973
	1.0	21	9	12	0.877
	1.1	24	10	14	0.85
	1.2	32	16	16	0.78
Pharmaceutical	0.25	0	0	0	0.862
	0.3	0	0	0	0.862
	**0.4**	**0**	**0**	**0**	**0.862**
	0.5	0	0	0	0.862
	0.6	0	0	0	0.862
	0.7	0	0	0	0.862
	0.8	0	0	0	0.862
	0.9	6	5	1	0.980
	1.0	18	15	3	0.784
	1.1	22	19	3	0.705
	1.2	23	20	3	0.588

**Table A5:** SODCM applied to 1-star review comments.

Dataset	$O_{s}$	$O_{D}$	$O_{I}$	$O_{C}$	Accuracy
Book	0.706	10	2	8	0.931
	0.8	24	4	20	0.956
	0.9	32	5	27	0.97
	**0.914**	**33**	**5**	**28**	**0.972**
	1.0	72	25	47	0.959
	1.1	110	55	55	0.892
	1.2	190	123	67	0.752
Electronics	0.809	2	1	1	0.929
	0.9	13	1	12	0.958
	**0.987**	**25**	**5**	**20**	**0.989**
	1.0	31	6	25	0.994
	1.1	89	30	59	0.844
	1.2	202	106	96	0.55
Entertainment	0.309	2	0	2	0.838
	0.4	3	1	2	0.84
	0.5	5	1	4	0.846
	**0.545**	**6**	**1**	**5**	**0.851**
	0.6	10	1	9	0.853
	0.7	16	1	15	0.865
	0.8	25	2	23	0.882
	0.9	48	7	41	0.925
	1.0	127	47	80	0.926
	1.1	185	64	121	0.818
	1.2	262	108	154	0.674
Grocery	0.357	2	0	2	0.933
	0.4	2	0	2	0.933
	0.5	6	3	3	0.939
	0.6	7	3	4	0.94
	**0.606**	**8**	**3**	**5**	**0.942**
	0.7	9	5	4	0.943
	0.8	13	6	7	0.949
	0.9	32	11	21	0.977
	1.0	121	77	44	0.89
	1.1	168	110	58	0.821
	1.2	229	153	76	0.731
Health Care	0.608	5	2	3	0.933
	0.7	8	3	5	0.938
	**0.795**	**14**	**4**	**10**	**0.948**
	0.8	14	4	10	0.948
	0.9	28	8	20	0.97
	1.0	92	46	46	0.925
	1.1	147	77	70	0.836
	1.2	244	148	96	0.679
Personal Care	0.312	5	0	5	0.922
	0.4	7	0	7	0.925
	0.5	11	0	11	0.931
	**0.575**	**12**	**0**	**12**	**0.933**
	0.6	13	1	12	0.933
	0.7	17	3	14	0.94
	0.8	24	3	21	0.951
	0.9	37	8	29	0.971
	1.0	137	86	51	0.876
	1.1	167	105	62	0.831
	1.2	231	156	75	0.733
Pharmaceutical	0.35	2	0	2	0.874
	0.4	2	0	2	0.874
	0.5	3	1	2	0.876
	**0.566**	**3**	**1**	**2**	**0.876**
	0.6	5	3	2	0.88
	0.7	11	6	5	0.891
	0.8	22	16	6	0.913
	0.9	42	31	11	0.951
	1.0	166	135	31	0.808
	1.1	211	172	39	0.722
	1.2	280	236	44	0.588
